# Application of agro-waste-mediated silica nanoparticles to sustainable agriculture

**DOI:** 10.1186/s40643-022-00496-5

**Published:** 2022-01-31

**Authors:** Pooja Goswami, Jyoti Mathur

**Affiliations:** Department of Bioscience and Biotechnology, Banasthali Vidyapith, Banasthali Tonk, 304022 Rajasthan India

**Keywords:** Agro-waste, Silica nanoparticles, Hydroponic, *Eruca sativa*

## Abstract

Use of green agronomic techniques for plant development and crop protection is essential for environmental sustainability. The current research investigates a more efficient and long-term technique of manufacturing silica nanoparticles (SiO_2_ NPs) from agricultural waste (sugarcane bagasse and corn cob). SiO_2_ NPs were synthesized by calcinations of waste residues in muffle furnace with varying temperatures (400–1000 °C)/2 h in the present of static air. Field emission scanning electron microscopy (FESEM), Fourier transmission infrared spectroscopy (FTIR), X-ray diffraction (XRD), and energy dispersive X-ray spectroscopy (EDX) were used to characterize SiO_2_ NPs and assessed for their antifungal activity simultaneously investigated the effects of various concentrations of produced SiO_2_ NPs on *Eruca sativa (E. sativa)* physiological and biochemical. With SiO_2_ NPs treatment at 1000 µg L^−1^ concentration, the seed germination rate was found to be up to 95.5%, and growth characteristics were enhanced compared to control. Accordingly, the ones treated with SiO_2_ NPs grew better than the control ones. The treatment of plant with SiO_2_ NPs (500 μg L^−1^) increased the protein content by 14.8 mg g^−1^, and chlorophyll level was also increased by 4.08 mg g^−1^ in leaves compared to untreated plant. Disc diffusion experiment was conducted to test the efficiency of SiO_2_ NPs against *Fusarium oxysporum* and *Aspergillus niger* for antifungal activities. Highest mycelia growth inhibition was obtained with 73.42% and 58.92% for *F. oxysporum* and *A. niger*, respectively. The result shows that the SiO_2_ NPs have a favorable effect on *E. sativa* growth and germination, enhancing plant production which helps to improve the sustainable agriculture farming and acting as a possible antifungal agent against plant pathogenic fungi.

**Graphical Abstract:**

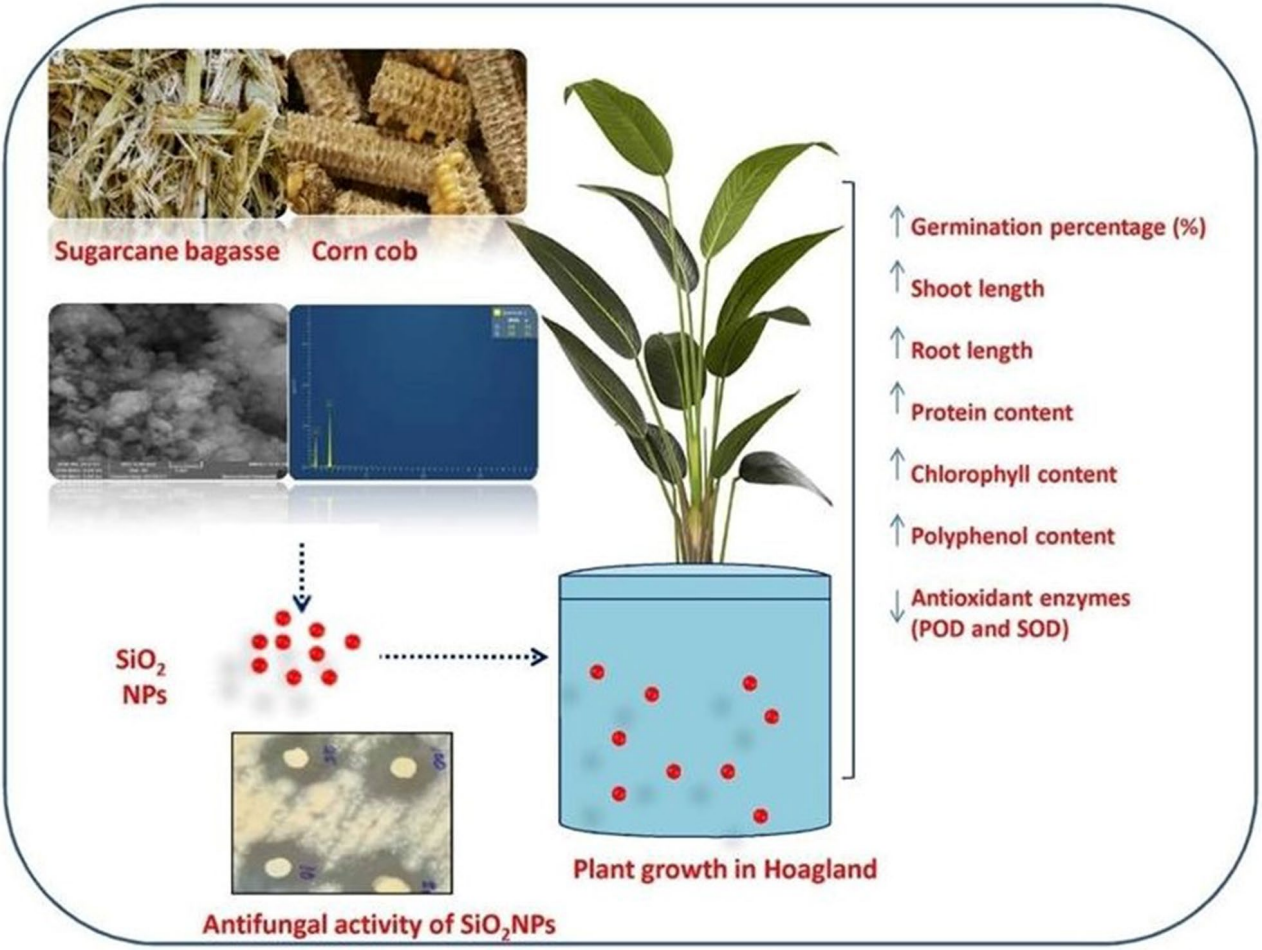

## Introduction

Nanomaterials potential uses are expanding in a variety of industries, including agriculture and biotechnology. Every year, agriculture-based industries generate massive amounts of trash, such as sugarcane bagasse, corncob, rice husk, wheat straw and discharge them into the environment. Dumping and burning of agro-wastes might behave as potent environmental pollutants. These wastes can be exploited as a starting point for the formation of useful nanomaterials. Silica nanoparticles (SiO_2_ NPs) mediated by agro-waste would be a unique concept. NPs and their derivatives are one-of-a-kind not only in terms of treatment approaches, but also in terms of physical and biological characteristics. However, research into the behavior of SiO_2_ NPs in agricultural applications is still in its infancy. However, advances in agricultural operations have necessitated the use of SiO_2_ NPs to improve stress tolerance and plant growth development (Reynolds et al. 2009). As a result, research have revealed that SiO_2_ NPs has a high positive response to biotic and abiotic stress, as well as metal toxicity such as copper, zinc, and iron (Tubana et al. 2016; Mostofa et al. 2021). Earlier, the antifungal activity of SiO_2_ NPs was well incorporated in the field of medical science. Fusarium and Aspergillus spp. are highly specialized in infecting crops, according to Aoudou et al. (2011). The application of SiO_2_ NPs to maize plants has indicated improved leaf transpiration rates under water stress (Kaya et al. 2006; Gao et al. 2006). Increased sensitivity to biotic and abiotic stresses is caused not only by a lack of necessary plant nutrients, but also by a decrease in silicon content in soil and plants (Ma and Yamaji 2008). SiO_2_ NPs undergoes polymerization in root tissues prior to transfer and deposition in the shoot sections (Debona et al. 2017). Because of their huge surface area and tiny size, SiO_2_ NPs are attracting a lot of attention in the agriculture industry. This ensures that SiO_2_ NPs diffuse well into root tissues (Hafez et al. 2021). SiO_2_ NPs (less than 20 nm) inhibited seed germination and growth of rice seedlings, according to Nair et al. (2011); however SiO_2_ NPs larger than 20 nm had good impacts on several plant parameters. Similarly, investigations found that tomato seedlings treated with SiO_2_ NPs had better seed germination (Siddiqui and Al-Whaibi 2014).

The existing field of nanobiotechnology is at the prime stage of development due to lack of execution of novel techniques in industrial scale and yet to be improved with innovative technologies.

Therefore, the present study investigated the antifungal potency of agro-waste-mediated SiO_2_ NPs by disc diffusion experiment and broth dilution assay. Comparative studies were also performed to analyze the impacts of these synthesized SiO_2_ NPs on physiological and biochemical aspects of taramira (*Eruca sativa*) seedlings (family: Brassicaceae) in terms of germination rate, morphological characteristics, chlorophyll content, protein and antioxidant enzymes. Agro-waste (sugarcane bagasse and corn cob) is more favorable than physical or chemical approaches for the production of SiO_2_ NPs since it is readily accessible, cost effective, eco-friendly, and practicable. This research will offer enough data to use SiO_2_ NPs to improve agricultural productivity.

## Material and methods

### Chemicals and materials

In the current study, analytical grade reagents were employed. Nitroblue tetrazolium (NBT), ethylenediamine tetra acetic acid (EDTA), dithiothreitol (DTT), polyvinylpolypyrrolidone (PVPP), Triton-X, and riboflavin were purchased from Sigma-Aldrich in India. For deionized water, a Millipore milli-Q system was used. Sugarcane bagasse (SB) was taken from the Daurala sugar mill (Uttar Pradesh) for the SiO_2_ NPs synthesis, while corn cob (CC) was collected from the local market of Jaipur, Rajasthan.

### Synthesis of SiO_2_ NPs

**Fig. 1:**
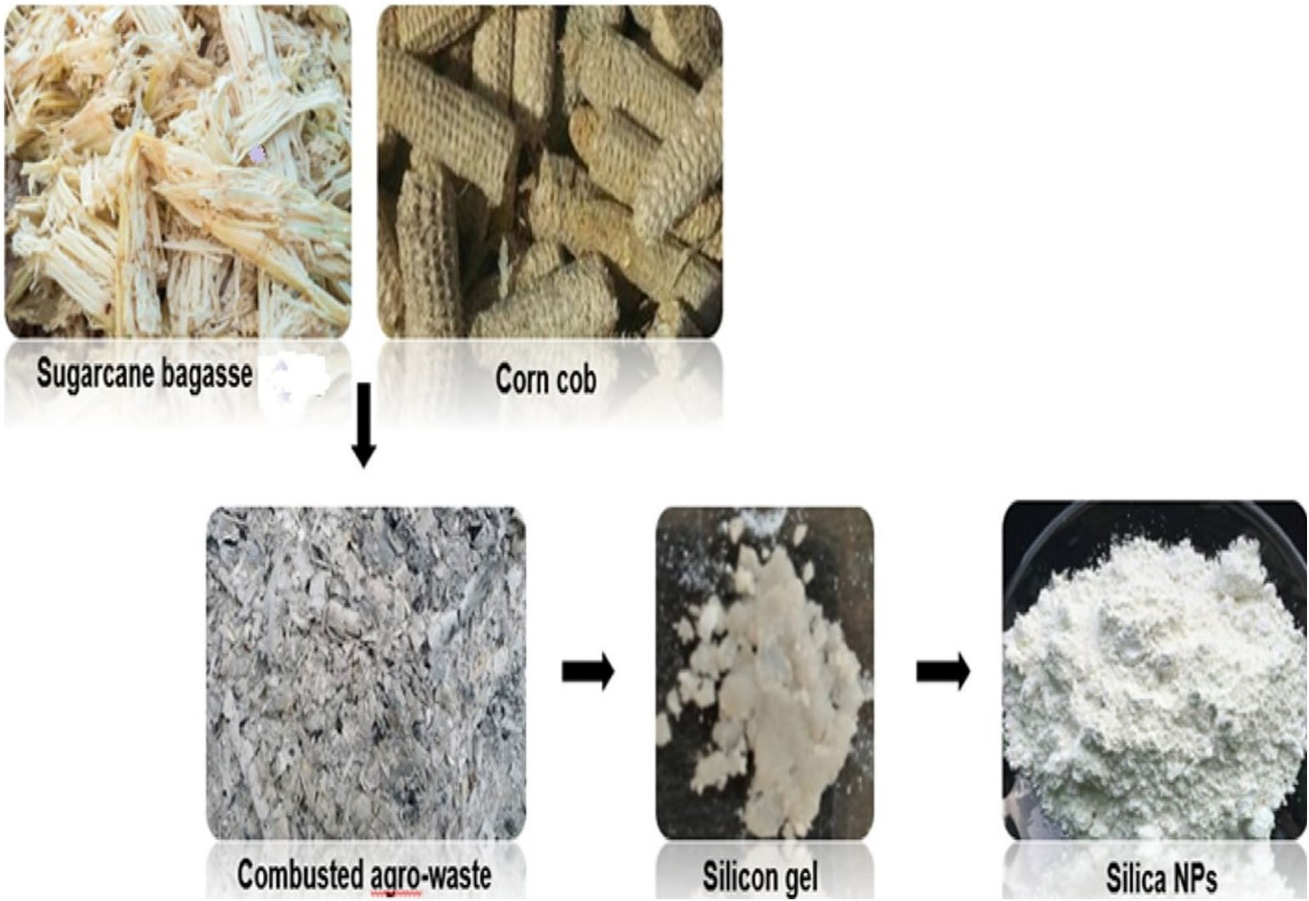
Schematic representation of SiO_2_ NPs synthesized from agro-waste (SB and CC)

SB and CC were cleaned and dried for 2 h at 110 °C. A 500 g of dry waste crushed into little crumbs. Waste residues were introduced into a muffle furnace for calcinations at different temperatures varying from 400 to 1000 °C at an interval of 200 °C for 2 h soaking time in static air. About 10 g of the resultant ash was agitated in 60 mL of a 1 N NaOH (sodium hydroxide) aqueous solution at 80 °C to dissolve silica and form sodium silicates (Na_2_SiO_3_). The clear solution was allowed to cool at room temperature, and the pH was maintained at 7 by applying 1 N HCl at constant stirring and then incubated for 12 h to commence gel formation. The synthesized gel was desiccated for 24 h at 80 °C to obtain xerogel (Fig. 1). To produce silica powder, the obtained xerogel was dried at 80 °C. (Sarangi et al. 2011; Chanadee and Chaiyarat 2016; Sethy et al. 2019).

### Characterization of SiO_2_ NPs

Various analytical methods were used to identify and validate the synthesized SiO_2_ NPs powder. Field emission scanning electron microscopy (FESEM) was used to examine the morphology of SiO_2_ NPs (model: MIRA3 TESCAN). Prior to FESEM, the samples were sputtered coated with a very thin layer of gold (Au). The elemental configuration of SiO_2_ NPs was determined using energy dispersive X-ray spectroscopy (EDX) associated with the FESEM. Aside from that, the crystalline structure of SiO_2_ NPs was investigated using X-ray diffraction (XRD), patterns (Bruker D8 Discover X-ray Diffraction). A small quantity of small (1 wt %) was scrupulously mixed with potassium bromide (KBr) pellet (FTIR grade) and a disc was prepared. Thereafter prepared pellet was measured through FTIR spectroscope (Bruker FTIR) have in the wave number region of 4000–400 cm^−1^ (Kumari and Khan 2017).

### Plant material

Sri Karan Narendra Agriculture University Jobner, Rajasthan, provided *E. sativa* seeds. The seeds were surface sterilized in a 10% sodium hypochlorite (NaClO) solution for 10 min before being germinated in petri plate (50 seeds/plate) with a double layer of filter paper.

### Hydroponic cultivation

Hoagland solution composed of multiple salts to provide a vital nutritional element. It is prepared by combining the macronutrients (g L^−1^) such as MgSO_4_.7H_2_O, KNO_3_, NH_4_NO_3_, KH_2_PO_4_, H_3_BO_4_, MnSO_4_.4H_2_O, CaCl_2_.2H_2_O and micronutrients as ZnSO_4_.7H_2_O, KI, CuSO_4_.5H_2_O, FeSO_4_.7H_2_O, Na_2_EDTA and Na_2_MoO_4_.2H_2_O. The entire investigation was carried out in a plant growth chamber under controlled conditions (photoperiod of 12 h at temperature 26 °C (± 2) and humidity 21% (± 2) and arranged as completely randomized block designs in replicates.

### Impact of SiO_2_ NPs on *E. sativa*

The effects of SiO_2_ NPs on *E. sativa* have been studied in terms of seed germination, physiological and biochemical properties. For the investigation, four different concentrations of SiO_2_ NPs were used: 100, 250, 500, and 1000 μg L^−1^ (Singh et al. 2015). The NPs suspension for treatment was sonicated for 30 min to obtain homogenous mixture. Surface sterilized 50 seeds were placed in their respective Petri dishes, and then a suspension of SiO_2_ NPs at each concentration was added to each Petri dish. Petri dishes were kept in the dark with regulated conditions for germination. Firstly, the germination rate was estimated based on the number of seeds germinated, and seeds with a root tip of 1 mm or greater were considered germinated. Root–shoot length (cm) and biochemical assays were carried out at 3, 6, and 9 days following seed germination.

### Polyphenol analysis

The polyphenol content was determined using the Bray and Thorpe (1954) method. A 0.1 g plant sample was extracted in 75% methanol. After adding 25% sodium carbonate, the absorbance was measured at 725 nm with a spectrophotometer.

### Determination of chlorophyll content

Li et al. (2016) demonstrated a technique for quantifying chlorophyll a and chlorophyll b in a leaf sample at 3, 6, and 9 days intervals. Seedlings were homogenized with 80% acetone and incubated overnight. The amount of chlorophyll in the sample was calculated as follows:

1 $${\text{Chl}}\,{\text{a}}\left( {\text{mg/L}} \right) = 12.72\left( {{\text{A}}_{663} } \right) - 2.59\left( {{\text{A}}_{645} } \right),$$

2 $${\text{Chl}}\,{\text{b}}\left( {\text{mg/L}} \right) = 22.88\left( {{\text{A}}_{645} } \right) - 4.67\left( {{\text{A}}_{663} } \right),$$

3 $${\text{Total chlorophyll}}\,{\text{content}}\left( {\text{mg/L}} \right) = {\text{Chl}}\,{\text{a}} + {\text{Chl}}\,{\text{b}}{.}$$

### Total protein

Total protein was calculated using the Bradford (1976) method at different concentration and time frames. This was done by mixing 100 μL of enzyme extract with 1 mL of Bradford solution and measuring absorbance at 595 nm.

### Antioxidant activity

The plant’s antioxidant potential was determined by monitoring the activity of various enzymes such as superoxide dismutase and peroxidase. Leaf samples (2.0 g) from non-treated and treated seedlings were rinsed and extracted in 10 mL extraction buffer (50 mM phosphate buffer (pH 7.0) containing 1 mM EDTA, 3 mM DTT, 5% w/v PVPP, 0.05% Triton-X). The crude extract was filtered using Whatman filter paper and centrifuged at 13,000 rpm for 30 min at 4 °C.

Peroxidase activity was measured using the method described by Güneş et al. (2019). 3 mL of solution comprising 0.5 mL of guaiacol solution in 0.1 mL of pH 7.0 sodium phosphate buffer, 0.3 mL of hydrogen peroxide, and 0.1 mL of enzyme extract were mixed for this. Peroxidase activity was measured using a spectrophotometer at 436 nm every 30 s for up to 3 min. The extinction coefficient (26.6 mM^−1^ cm^−1^) of guaiacol at 436 nm was used to calculate activity.

NBT in the presence of riboflavin was used to assess superoxide dismutase activity (Güneş et al. 2019). After mixing 50 μL enzymes extract with 1 mL NBT (50 M), 500 μL methionine (13 mM), 1 mL riboflavin (1.3 M), 950 μL (50 mM) phosphate buffer, and 500 μL EDTA (75 mM), the absorbance at 560 nm was measured.

### Microscopic analysis

The presence of SiO_2_ NPs in root, shoot, and leaf tissues was confirmed using a FESEM associated with EDX. The plant tissues were fixed in a 0.5 M phosphate buffer containing 2.5% glutaraldehyde, left overnight, and then dehydrated with a series of alcohol concentrations (Kumari and Khan 2018). Light microscopy was used to investigate plant tissues initially, and then SEM was used to examine them further. Aside from that, the existence of SiO_2_ NPs in root, shoot, and leaf tissues was confirmed by EDX analysis.

### Media for testing fungus and culture conditions

Strains of *Fusarium oxysporum* and *Aspergillus niger* were procured from MTCC, Chandigarh. Each fungal strain was sub-cultured at 27 °C in potato dextrose agar (Czerwinski and Szparaga 2015).

### Disc diffusion assay

Disc diffusion test was performed to evaluate the antifungal activity as described (Dhabalia et al. 2020). Sterile 6-mm disks were impregnated in the agar plates. Different concentrations of SiO_2_ NPs were pipetted onto sterile disks. A standard disk of Manocozeb was used as positive control for this study. Plates were then incubated at 30 °C for 24–48 h until a clear zone of inhibition was formed. The diameter of these zones was measured. Each test was conducted in triplicates to ensure reproducibility.

### Minimum inhibitory concentration (MIC)

MIC assay was carried out using the dilution method with slight modifications. 100 µL of SiO_2_ NPs of known concentration produced throughout sampling period were transferred into 96-well microtiter plates containing 100 µL of potato dextrose broth for fungal assay. Dilutions were performed by the twofold serial dilution method. Later, 100 µL of tested microorganisms were inoculated to all wells and the microtiter plates were incubated at 27 °C (48 h) for fungi. The minimum inhibitory concentration was determined as the lowest concentration of SiO_2_ NPs that inhibits the growth of microorganism (Basha and Ulaganathan 2002; Chan and Don 2012).

### Statistical analysis

The results were determined using the analysis of variance (ANOVA) test. Individual bars in the data represent the mean standard deviation of three replicates, followed by a ‘*’ signifying that the means were significantly different (*p* ≤ 0.05) using Tukey’s test.

## Results and discussion

### SiO_2_ NPs characterization

**Fig. 2:**
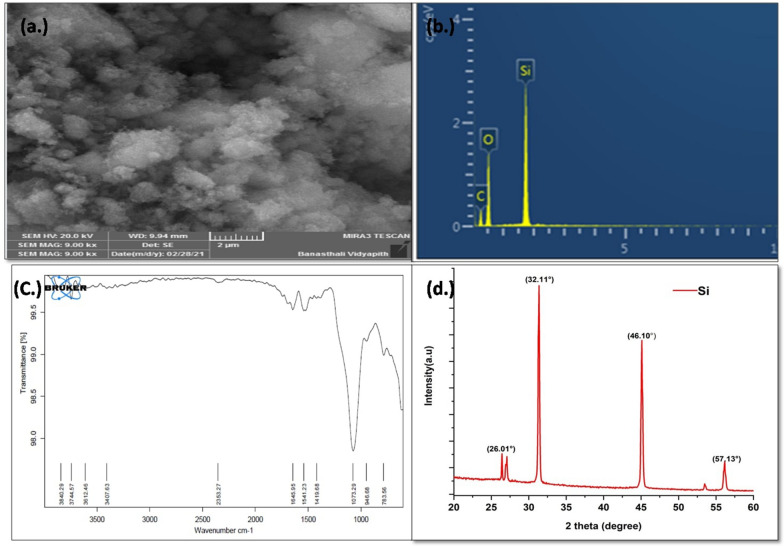
**a**–**d** shows characterization of synthesized SiO_2_ NPs. **a** FESEM, **b** EDX spectrum, **c** FTIR, and **d** XRD pattern

FESEM was used to examine the surface morphology of synthesized SiO_2_ NPs (Fig. 2a). The majority of NPs were found to be in a nano-agglomerated form with irregular SiO_2_ NPs shape. The EDX elemental spectrum revealed the presence of Si (43.84%), O (24.2%) and C (17.23%) in the component composition (Fig. 2b). The XRD pattern of SiO_2_ NPs is shown in Fig. 2c; strong diffraction peaks of SiO_2_ NPs were observed at 2*θ* = 36.01°, 32.11°, 46.10° and 57.13°. The diffraction peaks reported were similar to Suriyaprabha et al. 2012, and Chanadee and Chaiyarat (2016) which confirmed the crystallographic structure of SiO_2_ NPs. The Debye–Scherrer equation was used to calculate the average size of SiO_2_ NPs (Yew et al. 2016).

Debye–Scherrer equation is shown as:

4 $$D = k\lambda /\beta_{{{\text{hkl}}}} \cos \theta_{{{\text{hkl}}}} ,$$

where *D* is the crystallite size,* λ* is the X-ray wavelength of radiation for Cu Kά (0.154 nm), *β*_hkl_ is the full-width at half-maximum (FWHM), *k* is Scherrer constant (0.9) and *θ*_hkl_ is the diffraction angle. The average crystallite size of SiO_2_ NPs was 17.23 nm.

The chemical composition and functional groups of the produced SiO_2_ NPs were investigated using a FTIR spectrum. The 1073 cm^−1^ peak corresponds to the asymmetric stretching vibration and shear bands of Si–O–Si bonds. The symmetric stretching vibration of Si–O bonds is represented by the 800 cm^−1^ peak band (Palanivelu et al. 2014). The vibrational modifications of the silica gel network were disclosed by the peaks detected between 1090 and 799 cm^−1^ as evident at 1111.39 cm^−1^. Aside from that, a band at 1645 cm^−1^ was discovered, which matched to the adsorption of silanol OH groups (Fig. 2d) (Palanivelu et al. 2014).

### Effect of SiO_2_ NPs on *E. sativa*

**Fig. 3:**
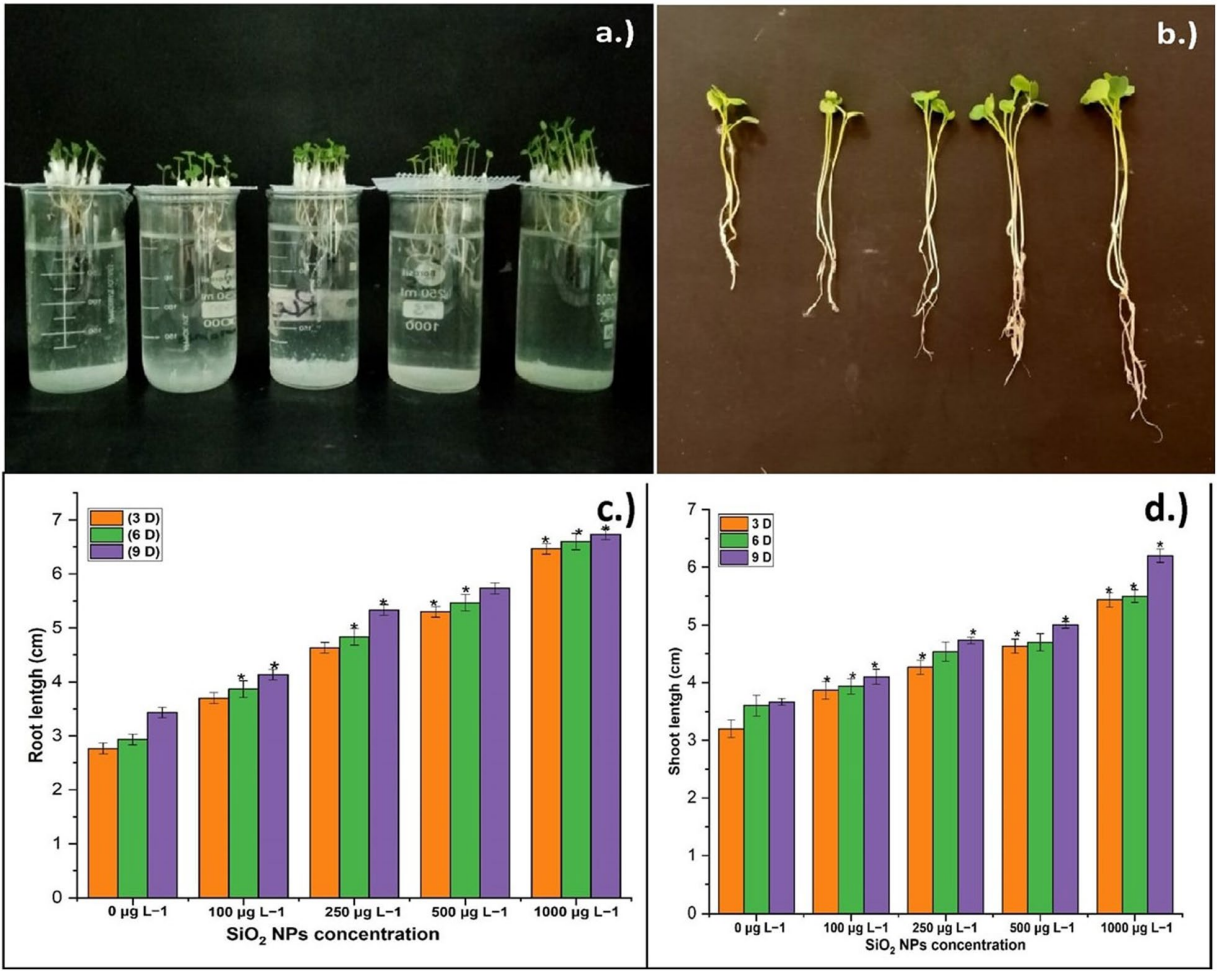
Effect of SiO_2_ NPs on *E. sativa* plant **a** and **c** root length and **b** and **d** shoot length at 9 days interval at 100–1000 μg L^−1^ concentration

The effects of SiO_2_ NPs on the majority of the evaluated morphological characteristics in *E. sativa* seedlings were favorable (Fig. 3a–d). Seed germination was measured for the observation by monitoring the radical presence. Control seedlings were those that had not been treated with SiO_2_ NPs. All plants treated with SiO_2_ NPs had significantly increased germination, shoot and root lengths. After 9 days of treatment with 1000 μg L^−1^ SiO_2_ NPs, the longest shoot length measured was 6.3 cm, while the shortest shoot length measured was 5.5 cm in the control (Fig. 3c). Simultaneously, the maximum root length at 1000 μg L^−1^ SiO_2_ NPs treatment was 6.6 cm, whereas the lowest root length in control seedlings was 6.3 cm (Fig. 3d). Overall, the results revealed that root and shoot length were somewhat increased at lower concentrations such as 250 μg L^−1^ and 500 μg L^−1^ and significantly increased at higher concentrations (1000 μg L^−1^). Similar findings were obtained for the treatment with SiO_2_ NPs, which improved the root–shoot length and growth of cucumber seedlings, however a minor drop was noted after a certain concentration. Maximum root–shoot lengths of 6.51 and 5.10 cm were recorded at 200 μg L^−1^ SiO_2_ NPs concentrations (Alsaeedi et al. 2019). The observations made above are consistent with the findings of Nair et al. (2011). This analysis revealed that using FITC-labeled SiO_2_ NPs enhanced rice seedling germination. As a result, based on the outcome of SiO_2_ NPs treatment, we may imagine their direct and indirect engagement in plant growth (Fig. 3a-b) via an increase in seed germination qualities.

### Polyphenol content

**Fig. 4:**
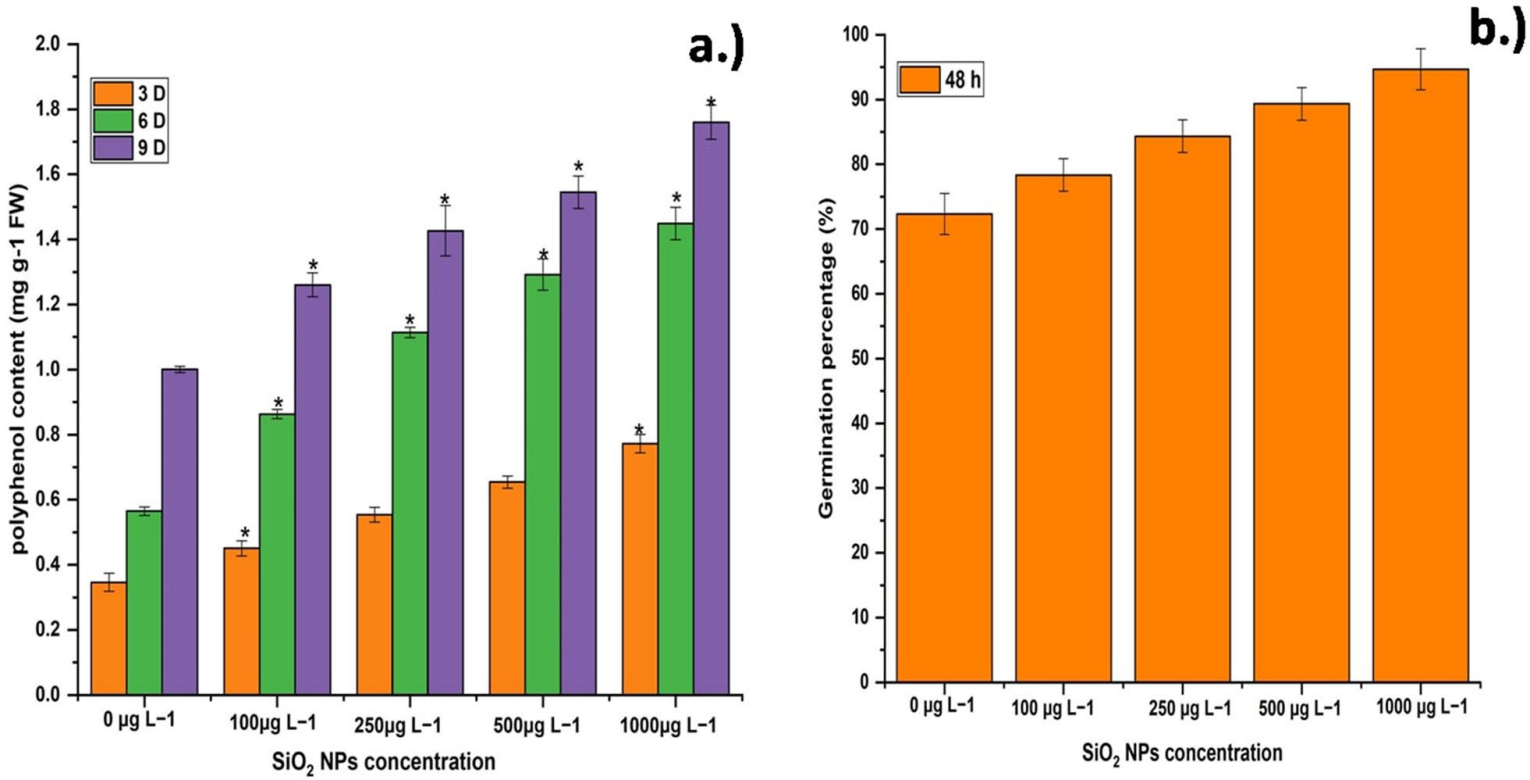
Effect of different concentrations of SiO_2_ NPs on *E. sativa* **a** germination percentage at 48 h with concentration 100, 250, 500 and 1000 μg L^−1^ of SiO_2_ NPs and **b** polyphenol content with same treatment

Phenols are important defense compounds that protect plants from a variety of stresses because they absorb and deactivate free radicals and decompose peroxides (Shah et al. 2010). The effect of SiO_2_ NPs on the polyphenol content of treated *E. sativa* seedlings is demonstrated (Fig. 4b). The treatment of varied concentrations of SiO_2_ NPs enhanced the polyphenol content linearly. At 9 days intervals, the highest polyphenol content was recorded at 1000 μg L^−1^ SiO_2_ NPs therapy while the lowest was reported at 100 μg L^−1^ SiO_2_ NPs treatment. Similarly, SiO_2_ NPs treatments boosted the accumulation of phenolic compounds in leaf epidermis compared to untreated leaves. The mechanism of SiO_2_ NPs induced phenols may be due to the accumulation of insoluble silica NPs in the epidermis, which induces the enrichment of constitutional phenols in epidermal cells due to their super high adsorption surface (Li et al. 2004).

### Determination of chlorophyll and protein content

**Fig. 5:**
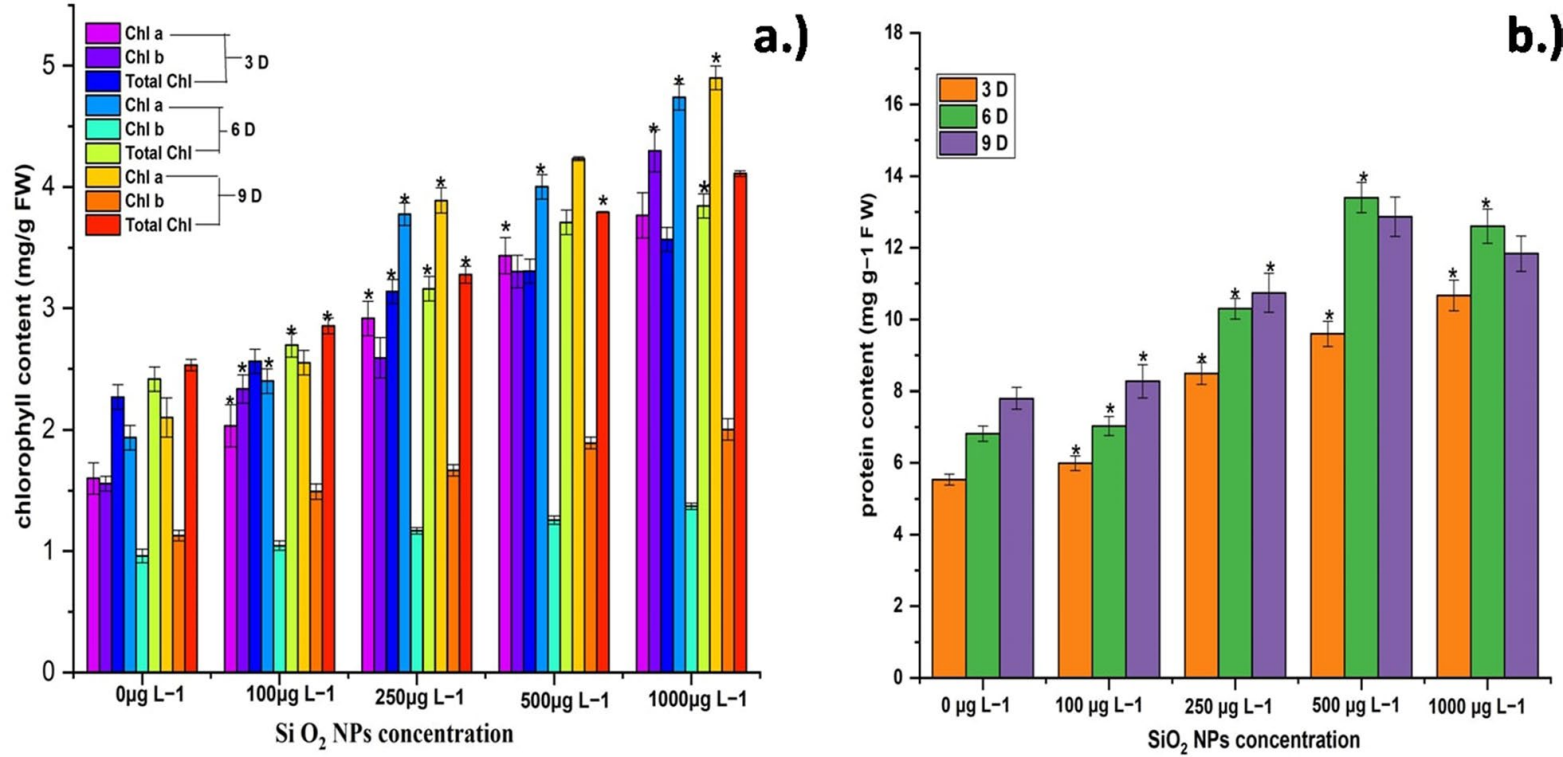
**a** Chlorophyll a (Chl a), chlorophyll b (Chl b) and total chlorophyll content and **b** protein content in fresh leaves of *E. sativa* with 100 μg L^−1^, 250 μg L^−1^, 500 μg L^−1^ and 1000 μg L^−1^ of SiO_2_ NPs after 9 days hydroponic cultivation

Leaves were obtained from treated and control pots, revealing that the chlorophyll a and b contents increased considerably with increasing SiO_2_ NPs concentrations and time intervals (Fig. 5a). Chlorophyll a, b, and total content were found to be highest at 1000 μg L^−1^ SiO_2_ NPs concentration and lowest at 100 μg L^−1^ SiO_2_ NPs concentration. El-Serafy (2019) observed comparable results in his study. Sun et al. (2016) found that mesoporous SiO_2_ NPs enhanced chlorophyll a, b, and total concentrations, which supported these findings. The results showed an increase in total protein content at 3, 6, and 9 days for all SiO_2_ NPs treatments (100, 250, 500, and 1000 μg L^−1^). The reported total protein content with 100 μg L^−1^ treatment, on the other hand, revealed no significant variation as compared to control pots (Fig. 5b). In comparison to 100, 250, and 1000 μg L^−1^ SiO_2_ NPs treatments, 500 μg L^−1^ SiO_2_ NPs treatment yielded the highest protein content. Protein content was reduced at the maximum SiO_2_ NPs concentration of 1000 μg L^−1^, demonstrating the harmful effect of SiO_2_ NPs over a specific concentration. Sun et al. (2016) showed similar results of protein content increase up to a specific threshold.

### Impact on oxidative stress

**Fig. 6:**
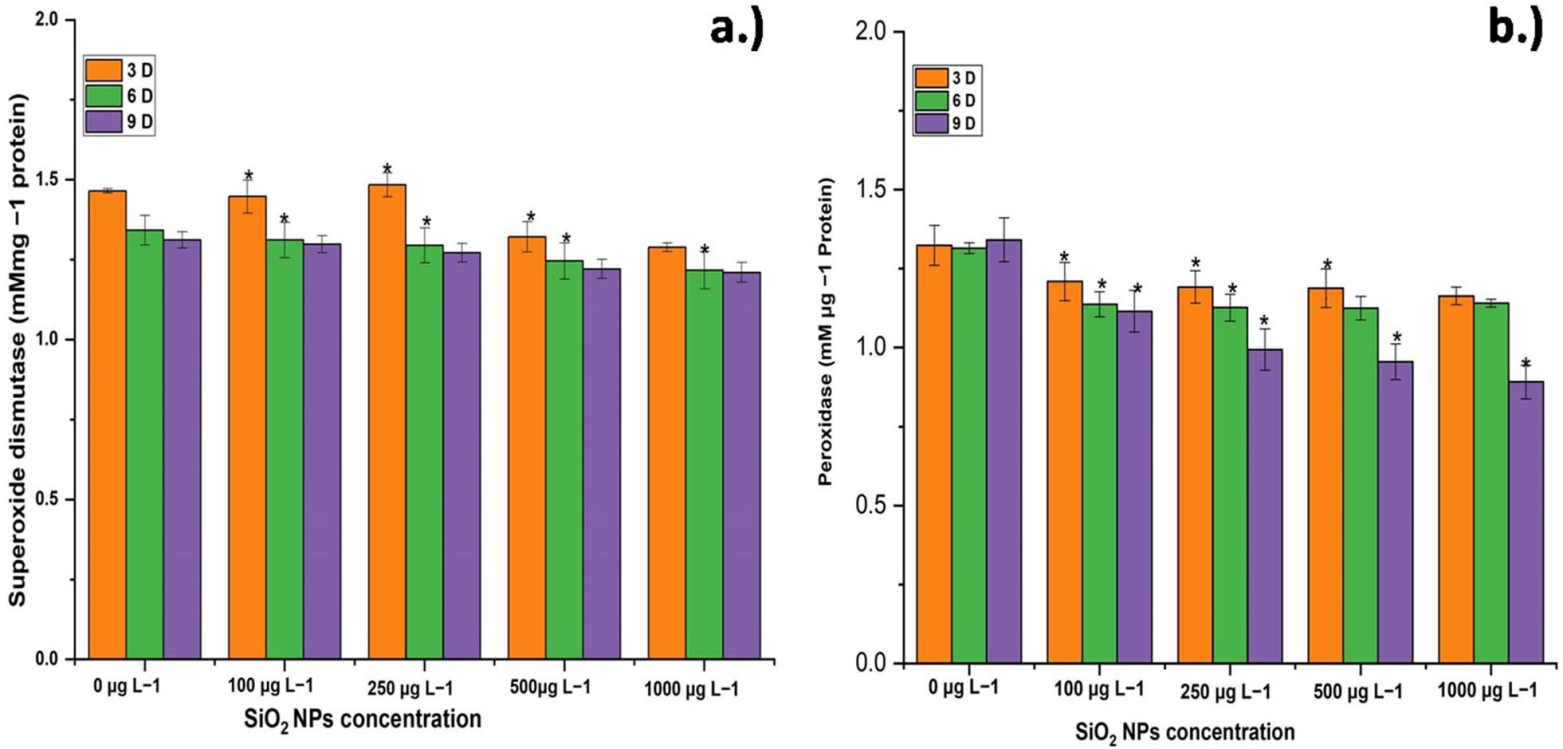
Antioxidant activity **a** peroxidase and **b** superoxide dismutase of *E. sativa* with 100, 250, 500 and 1000 μg L^−1^ of SiO_2_ NPs after 9 days

Under oxidative stress, antioxidant enzymes and metabolites exert a significant control on the development of reactive oxygen species (ROS) and their fatal effects. The antioxidant capacity of the seedlings increased as the amounts of SiO_2_ NPs increased, as measured by the activity of antioxidant enzymes. In contrast to earlier research, the activity of both antioxidant enzymes, peroxidase and superoxide dismutase, was lowered with increasing SiO_2_ NPs treatments, even when the treatment method was the same. Peroxidase activity reduced when SiO_2_ NPs treatments increased, according to spectrophotometric analysis (Fig. 6a). All SiO_2_ NPs treatments (100–1000 μg L^−1^) reduced peroxidase activity. The activity of superoxide dismutase and peroxidase reduced as the concentration of SiO_2_ NPs increased from 100 to 1000 μg L^−1^ (Fig. 6b). This reduction might be due to the better growing medium and nutrition given by SiO_2_ NPs. Cucumber seedlings treated with SiO_2_ NPs showed a reduction in the quantity of ROS species (i.e., H_2_O_2_) in a comparable research (Alsaeedi et al. 2019). A linear reduction in peroxidase activity was found as a result of increasing the dosage of applied SiO_2_ NPs. The soil treatment (S 200) produced the maximum peroxidase activity, whereas the foliar treatment of 200 mg L^−1^ produced the lowest peroxidase activity when compared to the soil treatment of the same dose (Attia and Elhawat 2021).

### Microscopic studies

**Fig. 7:**
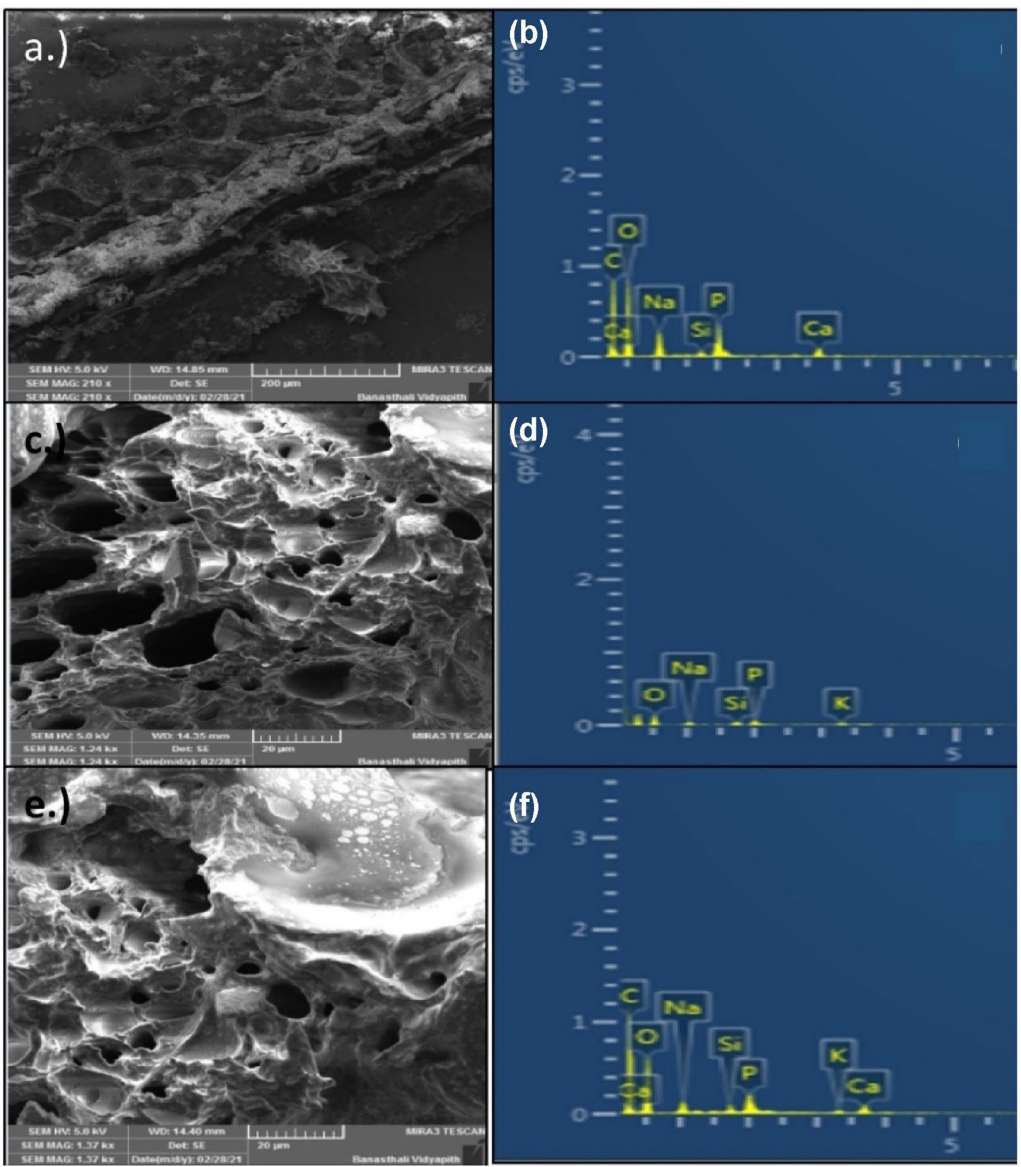
**a** and **b** shows the FESEM and EDX spectrum of leaf tissues, **c** and **d** shoot tissue and **e** and **f** root tissues of *E. sativa seedling* at 1000 μg L^−1^ SiO_2_ NPs treatment after 9 days

FESEM images of transverse sections (T.S.) of shoot and root tissues at 1000 μg L^−1^ revealed SiO_2_ NPs uptake and accumulation in leaf, shoot, and root tissues of treated seedlings (Fig. 7). The existence of NPs was confirmed after 9 days of SiO_2_ NPs therapy (Fig. 7c and e). The presence of SiO_2_ NPs was also visible in FESEM images of leaf tissues (Fig. 7a). The presence of SiO_2_ NPs peaks in leaf, shoot, and root tissues was confirmed by EDX analysis for further validation (Fig. 7b, d, f).

### Antifungal activity

The disc diffusion experiment was used to assess the antifungal efficacy of SiO_2_ NPs against *F. oxysporum* and *A. niger* mycelia on potato dextrose agar plates. A control plate with no SiO_2_ NPs was maintained independently for both fungal strains. *Fusarium oxysporum* and *A. niger* were both inhibited by SiO_2_ NPs produced from agro-waste (SB and CC), but no zone of inhibition was identified in control plates. At 1000 μg SiO_2_ NPs concentration, the maximum percent inhibition reported in *F. oxysporum* and *A. niger* was 73.42 ± 1.14 and 58.92 ± 3.49, respectively (Table 1). The minimum inhibitory concentrations for *F. oxysporum* and *A. niger* were 3.1 and 6.3 μg mL^−1^, respectively. Similarly, higher antifungal effectiveness of mesoporous SiO_2_ NPs against *Alternaria solani* in tomato plants has been found (Derbalah et al. 2018). The study demonstrated the highest inhibitory effectiveness of about 95% for both fungus *F. oxysporum* and *A. niger* (Akpinar et al. 2017).

**Table 1:** Percentage (%) of growth inhibition of *F. oxysporum* and *A. niger* by SiO_2_ NPs

Fungus	SiO_2_ NPs content					
	1000 μg	500 μg	250 μg	100 μg	Standard	Control
*Fusarium oxysporum*	73.42 ± 1.14	64.28 ± 2.30	61.30 ± 1.69	53.1 ± 1.52	M (97.67 ± 0.0 M)	0.00 ± 0.00
*Aspergillus niger*	58.92 ± 3.49	51.1 ± 2.79	43.7 ± 1.90	41.5 ± 1.58	F (100)	0.00 ± 0.00

Each value represented in table are means ± SD (*N* = 3), 0.00: indicates no inhibition

M, Manocozeb

## Conclusion

The current work demonstrates an efficient and economical green approach for producing SiO_2_ NPs from SB and CC. At 3, 6, and 9 days intervals, SiO_2_ NPs doses of 100, 250, 500, and 1000 μg L^−1^ were administered. SiO_2_ NPs applied to *E. sativa* seedlings improved not only plant biometrics and physiology, but also served as an antifungal agent. SiO_2_ NPs inhibited *F. oxysporum* and *A. niger* with maximal inhibition percentages of 73.42 and 58.92, respectively. Many processes, such as plant interaction with SiO_2_ NPs and their cellular and molecular activities, necessitate further extensive investigation on all of these concerns. As a result, SiO_2_ NPs might be useful in agriculture sectors as fungicides and fertilizers.

## Data Availability

All data generated or analyzed during this study are included in this article.

## Abbreviations

SiO_2_NPs: Silica nanoparticles
SEM: Scanning electron microscope
FTIR: Fourier transmission infrared spectroscopy
XRD: X-ray diffraction
EDX: Energy dispersive X-ray
SBA: Sugarcane bagasse
MIC: Minimum inhibitory concentration
